# Consumer Perceptions of Safety Information in Direct-to-Consumer Print Advertisements for Alzheimer Drugs

**DOI:** 10.1001/jamanetworkopen.2024.31110

**Published:** 2024-08-30

**Authors:** Jenny Markell, Ilina Odouard, Gerard F. Anderson, Michael J. DiStefano

**Affiliations:** 1 Department of Health Policy and Management, Johns Hopkins Bloomberg School of Public Health, Baltimore, Maryland; 2 Center for Pharmaceutical Outcomes Research, Department of Clinical Pharmacy, Skaggs School of Pharmacy and Pharmaceutical Sciences, University of Colorado Anschutz Medical Campus, Aurora

## Abstract

This survey study assesses the effectiveness of drug advertisement to shape perception of the benefits and risks of and personal willingness to use and recommend an advertised drug.

## Introduction

The US Food and Drug Administration (FDA) requires that direct-to-consumer advertising (DTCA) describe a drug’s benefits and risks in a balanced way and recommends presenting effectiveness and risk information quantitatively to improve consumer comprehension.^1,2^ Print product-claim advertisements must include a brief summary of all risks, but there is no requirement that every risk be prominently presented or severe risks be highlighted.^3^

Presentation of risk information in DTCA is of particular concern for lecanemab, a new Alzheimer disease drug with an FDA black box warning due to the possibility of severe brain swelling and bleeding.^4^ This survey study investigates the association of displaying severe adverse effects in print DTCA, comparing the presentations both qualitatively and quantitatively, with perceptions of a drug and willingness to take that drug.

## Methods

Data were collected via NORC's Omnibus survey, which includes a nationally representative sample of NORC's AmeriSpeak panel (see eMethods in Supplement 1). Demographic information was self-reported when participants joined the panel and included routine data such as race and ethnicity. Consent was obtained prior to survey participation. The survey was administered online from November 30 to December 4, 2023. The Johns Hopkins Bloomberg School of Public Health and NORC Institutional Review Boards deemed this study exempt from ethics review. We followed the AAPOR reporting guideline.

We adapted the design of a previous study investigating consumer preferences in DTCA.^5^ Participants were randomly assigned to see 1 of 4 print advertisements (eAppendix in Supplement 1) for a fictitious Alzheimer drug, with risk and benefit information based on published data for lecanemab.^6^ Advertisement 1 included qualitatively described benefits and common adverse effects, advertisement 2 added qualitatively described severe adverse effects, advertisement 3 included quantitatively described benefits and common adverse effects, and advertisement 4 added quantitatively described severe adverse effects. After viewing 1 advertisement, participants completed a survey using 5-point Likert scales to report their perceptions of the drug’s benefits, risks, and overall balance of benefits and risks as well as likelihood of asking a physician about the drug and using the drug.

We compared mean (SD) scores using unpaired, 2-tailed *t* tests and accounted for survey weights in Stata 17 (StataCorp LLC). We conducted a sensitivity analysis to compare medians using unweighted Wilcoxon rank sum tests. Two-sided *P* < .05 indicated statistical significance.

## Results

The survey had a 16% completion rate. Among 1007 survey respondents, 232 (23.0%) were 65 years or older, 516 were females (51.3%), and 491 were males (48.7%). Table 1 provides respondents’ demographic characteristics.

**Table 1.** Demographic Characteristics of Study Participants

Characteristics	Participants, Weighted No. (%)^a^
Age, y	
18-24	117 (11.6)
25-34	176 (17.4)
35-44	180 (17.9)
45-54	126 (12.5)
55-64	181 (17.9)
65-74	158 (15.6)
≥75	69 (6.9)
Sex	
Male	491 (48.7)
Female	516 (51.3)
Race and ethnicity^b^	
Asian or Other Pacific Islander, non-Hispanic	67 (6.7)
Black, non-Hispanic	122 (12.1)
Hispanic	175 (17.3)
White, non-Hispanic	618 (61.4)
≥2 races, non-Hispanic^c^	20 (2.0)
Other, non-Hispanic^c^	6 (0.6)
Educational level	
<High school	92 (9.2)
High school diploma or equivalent	292 (29.0)
Some college or associate’s degree	265 (26.3)
Bachelor’s degree	226 (22.4)
Postgraduate study or professional degree	133 (13.2)
Household income, $	
<10 000	41 (4.0)
10 000 to <20 000	71 (7.1)
20 000 to <30 000	96 (9.5)
30 000 to <40 000	77 (7.6)
40 000 to <50 000	68 (6.8)
50 000 to <75 000	184 (18.2)
75 000 to <100 000	154 (15.3)
100 000 to <150 000	185 (18.4)
≥150 000	132 (13.1)

^a^ Percentages may not add up to 100% due to rounding. Weighted demographic statistics were within 0.3 percentage points of benchmark values taken from the US Census Bureau Current Population Survey (March 2023) for age (18-24, 25-64, ≥65 years), sex, race and ethnicity (non-Hispanic Black, Hispanic, White, all other), and educational level (<high school, some college, ≥Bachelor degree).

^b^ Race and ethnicity were self-reported when participants joined the AmeriSpeak panel.

^c^ The category ≥2 races, non-Hispanic indicated that participants chose more than 1 of the race categories. Other category included any other races not listed.

As quantitative replaced qualitative description and information about severe adverse effects was added to the advertisement, respondents perceived the drug to have greater risks, be less effective, or have more risks than benefits and were less likely to ask their physician about the drug or consider using the drug or recommend it to a family member (Table 2). Results were not significant among those who viewed all quantitative advertisements: 1 group with vs 1 group without severe adverse effects (advertisement 4 vs advertisement 3). However, in the sensitivity analysis, those who viewed an advertisement with quantitative description and severe adverse effects were significantly more likely to perceive the drug as riskier.

**Table 2.** Survey Responses About Direct-to-Consumer Advertising^a^

Questions	Mean (SD) score								
	Overall sample, mean (SD) (n = 1007)	Advertisement 1: qualitative benefits and common adverse effects (n = 235)^b^	*P* value	Advertisement 2: qualitative benefits and common + severe adverse effects (n = 266)^c^	*P* value	Advertisement 3: quantitative benefits and common adverse effects (n = 233)^d^	*P* value	Advertisement 4: quantitative benefits and common + severe adverse effects (n = 273)^e^	*P* value
1. How effective do you think Altana would be for you or a family member? (1 [not at all effective] to 5 [very effective])	2.69 (0.84)	3.05 (0.80)	.007	2.77 (0.81)	.003	2.45 (0.83)	<.001	2.45 (0.79)	.97
2. How risky do you think Altana would be for you or a family member? (1 [not at all risky] to 5 [very risky])	3.24 (0.85)	2.90 (0.78)	<.001	3.38 (0.77)	.53	3.24 (0.85)	.004	3.44 (0.89)	.09
3. Thinking overall about the risks and benefits, would you say that Altana has… (1 [much more risks than benefits] to 5 [much more benefits than risks])	2.87 (1.12)	3.49 (0.99)	<.001	2.94 (1.09)	.001	2.48 (0.98)	<.001	2.52 (1.12)	.80
4. How likely would you be to ask your doctor or encourage your family member to ask their doctor about Altana? (1 [not at all likely] to 5 [very likely])	2.62 (1.10)	3.21 (1.09)	<.001	2.69 (1.00)	<.001	2.35 (1.08)	<.001	2.22 (0.96)	.42
5. How likely are you to use Altana or recommend that a family member use Altana? (1 [not at all likely] to 5 [very likely])	2.44 (1.01)	2.93 (0.97)	<.001	2.49 (0.98)	.008	2.19 (0.94)	<.001	2.14 (0.94)	.68

^a^ Nonresponders were dropped by question, which was less than 0.2% per question. Sensitivity analysis was conducted to compare medians using unweighted Wilcoxon rank sum tests. Findings were largely similar, although in the sensitivity analysis, those who viewed quantitative severe adverse effects were significantly more likely to perceive the drug as riskier.

^b^ Two-sample *t* tests incorporating study weights were conducted to assess whether the mean value of the column was statistically different from the mean value of advertisement 2 column.

^c^ Two-sample *t* tests incorporating study weights were conducted to assess whether the mean value of the column was statistically different from the mean value of advertisement 4 column.

^d^ Two-sample *t* tests incorporating study weights were conducted to assess whether the mean value of the column was statistically different from the mean value of advertisement 1 column.

^e^ Two-sample *t* tests incorporating study weights were conducted to assess whether the mean value of the column was statistically different from the mean value of advertisement 3 column.

## Discussion

Prominently including severe adverse effects in DTCA was associated with consumers perceiving the advertised drug as riskier. Providing quantitative information on benefits and adverse effects also was a factor in consumer perception of the drug as riskier. For Alzheimer drugs, the FDA should consider requiring that any severe adverse effects listed in the black box warning be prominently displayed in DTCA.

This study has limitations. The survey had a low completion rate, although it adjusted for nonresponse with weighting and used a 2-stage sampling design to include a representative sample of US households. Additionally, findings may not apply to other drugs.

## Notes

Supplement 1.
eMethods
**eAppendix.** Ads 1-4

Supplement 2.
Data Sharing Statement
