# Supplementary material for: Consumer Perceptions of Safety Information in Direct-to-Consumer Print Advertisements for Alzheimer Drugs
Source: JAMA Netw Open. 2024 Aug 30;7(8):e2431110. doi: 10.1001/jamanetworkopen.2024.31110 (PMC11364991; doi:10.1001/jamanetworkopen.2024.31110)
Supplement: Supplement 1. — eMethods eAppendix. Ads 1-4 [file jamanetwopen-e2431110-s001.pdf]

Supplemental Online Content

Markell J, Odouard I, Anderson GF, DiStefano MJ. Consumer perceptions of safety information in direct-to-consumer print advertisements for Alzheimer drugs. *JAMA Netw Open*. 2024;7(9):e2431110. doi:10.1001/jamanetworkopen.2024.31110

eMethods

eAppendix. Ads 1-4

This supplemental material has been provided by the authors to give readers additional information about their work.

© 2024 Markell J et al. *JAMA Network Open*

eMethods

For more information on NORC's AmeriSpeak panel, please see the following reference:

Technical Overview of the AmeriSpeak Panel: NORC's Probability-Based Household Panel. NORC at the University of Chicago; 2022. Accessed January 5, 2024. https://amerispeak.norc.org/content/dam/amerispeak/research/pdf/AmeriSpeak%20Technical%20Overview%202019%2002%2018.pdf

© 2024 Markell J et al. *JAMA Network Open*

eAppendix. Ads 1-4

Ad 1

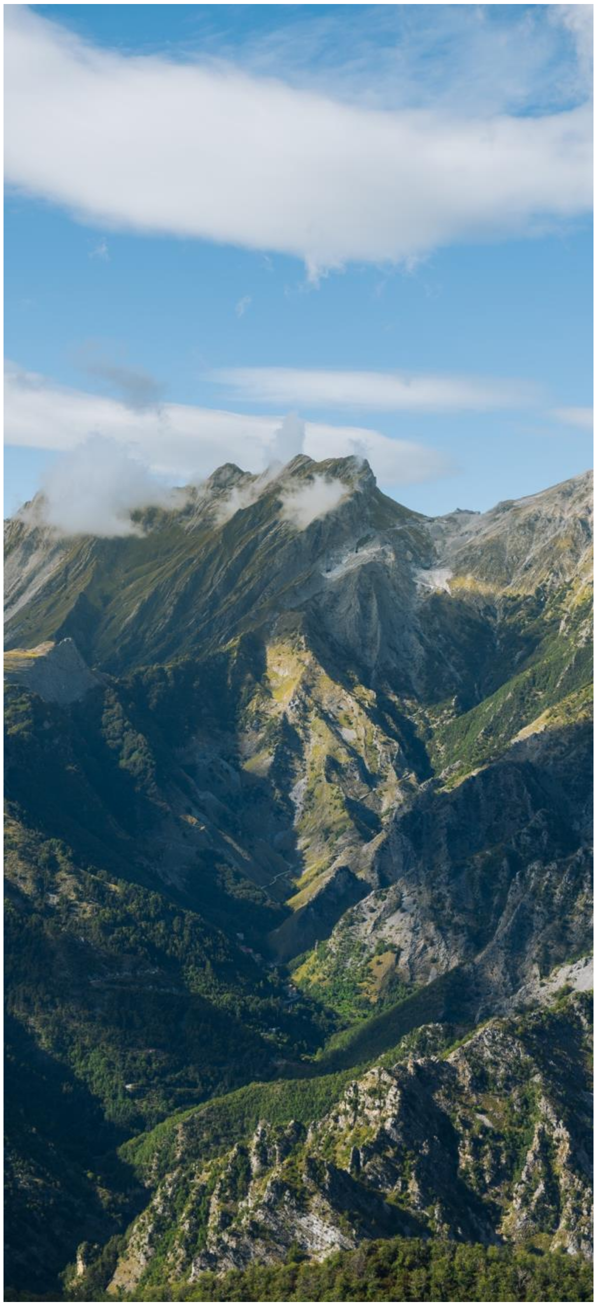

Altana

altenemab (injection for intravenous use)

Altana is a prescription medicine that is used to treat adults with mild cognitive impairment or mild dementia due to Alzheimer's disease

Altana was shown to slow progression of early Alzheimer's disease, helping people remember, solve problems, and complete daily activities for longer

Common side effects of **Altana** include fever, flu-like symptoms, and nausea

You are encouraged to report negative side effects of prescription drugs to the FDA. Visit www.fda.xyz/reportdrugs, or call 1-800-FDA-ABCD

Ask your doctor if **Altana** is right for you

Ad 2

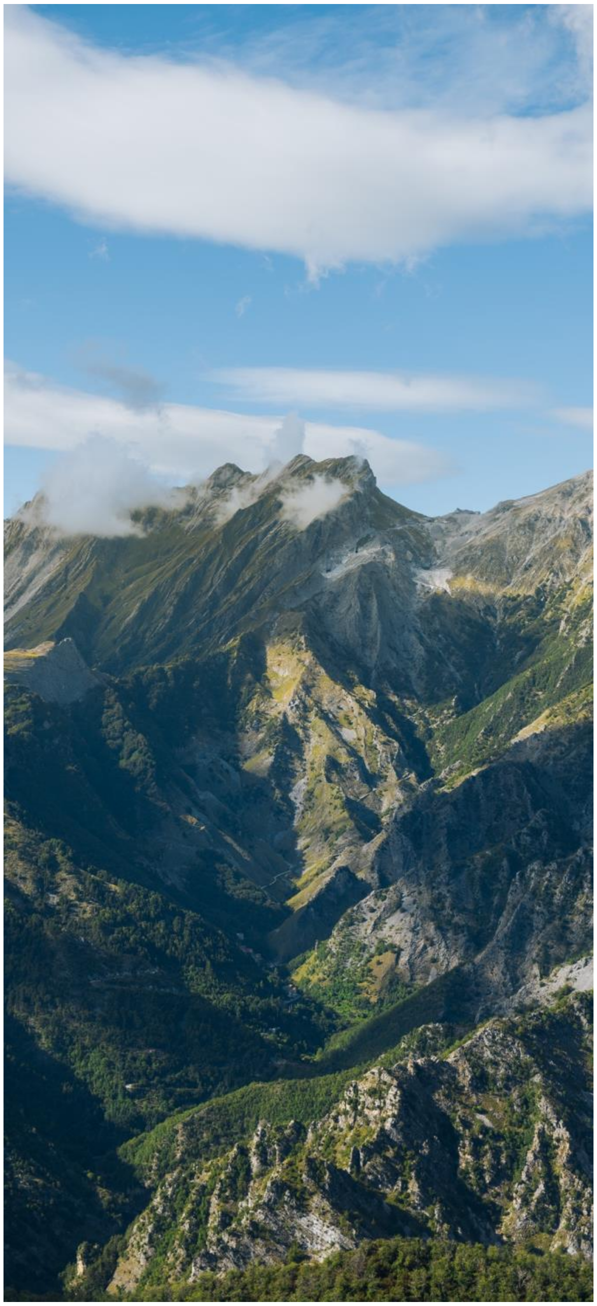

Altana

altenemab (injection for intravenous use)

Altana is a prescription medicine that is used to treat adults with mild cognitive impairment or mild dementia due to Alzheimer's disease

Altana was shown to slow progression of early Alzheimer's disease, helping people remember, solve problems, and complete daily activities for longer

Common side effects of **Altana** include fever, flu-like symptoms, and nausea. Some people experience **life threatening** symptoms such as swelling or bleeding in the brain

You are encouraged to report negative side effects of prescription drugs to the FDA. Visit www.fda.xyz/reportdrugs, or call 1-800-FDA-ABCD

Ask your doctor if **Altana** is right for you

© 2024 Markell J et al. *JAMA Network Open*

Ad 3

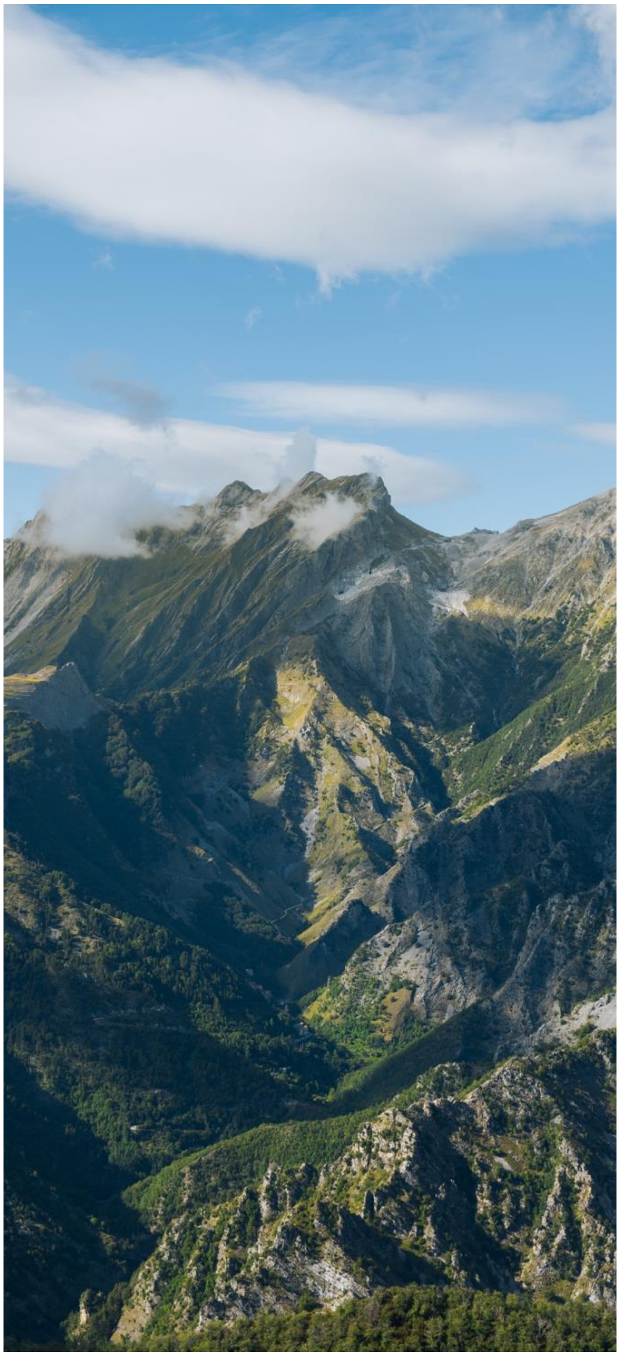

Altana

altenemab (injection for intravenous use)

Altana is a prescription medicine that is used to treat adults with mild cognitive impairment or mild dementia due to Alzheimer's disease

In an 18-month clinical trial, people taking **Altana** declined in cognitive function on average by 1.21 points on an 18-point scale (7%), while people not taking the drug declined in cognitive function on average by 1.68 points on an 18-point scale (9%)

In a clinical trial, 237 out of 898 (26%) people taking **Altana** experienced side effects such as fever, flu-like symptoms, and nausea compared to 66 out of 897 (7%) in people not taking the drug

You are encouraged to report negative side effects of prescription drugs to the FDA. Visit www.fda.xyz/reportdrugs, or call 1-800-FDA-ABCD

Ask your doctor if **Altana** is right for you

© 2024 Markell J et al. *JAMA Network Open*

Ad 4

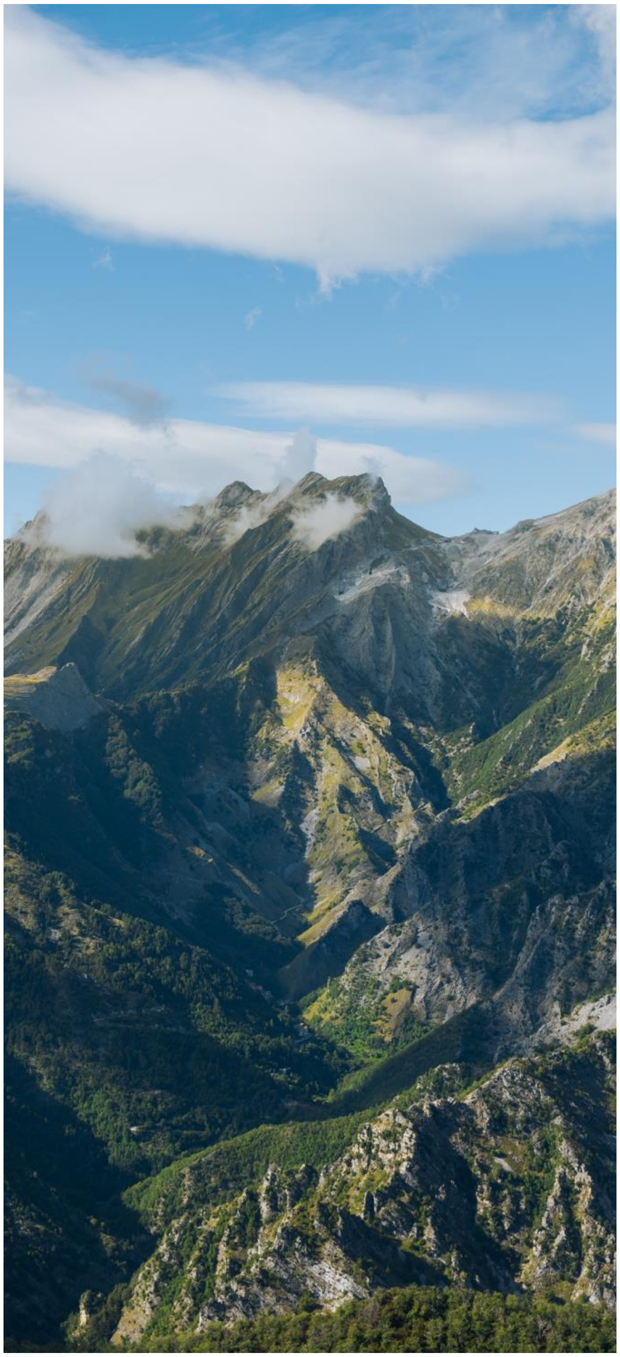

Altana

altenemab (injection for intravenous use)

Altana is a prescription medicine that is used to treat adults with mild cognitive impairment or mild dementia due to Alzheimer's disease

In an 18-month clinical trial, people taking **Altana** declined in cognitive function on average by 1.21 points on an 18-point scale (7%), while people not taking the drug declining in cognitive function on average by 1.68 points on an 18-point scale (9%)

In a clinical trial, 237 out of 898 (26%) people taking **Altana** experienced side effects such as fever, flu-like symptoms, and nausea compared to 66 out of 897 (7%) in people not taking the drug. 31 out of 898 people (3%) had **life threatening** symptoms of swelling or bleeding in the brain compared to 2 out of 897 (0.2%) in people not taking the drug

You are encouraged to report negative side effects of prescription drugs to the FDA. Visit www.fda.xyz/reportdrugs, or call 1-800-FDA-ABCD

Ask your doctor if **Altana** is right for you

© 2024 Markell J et al. *JAMA Network Open*
