# Supplementary material for: Consumer Perceptions of Safety Information in Direct-to-Consumer Print Advertisements for Alzheimer Drugs
Source: JAMA Netw Open. 2024 Aug 30;7(8):e2431110. doi: 10.1001/jamanetworkopen.2024.31110 (PMC11364991; doi:10.1001/jamanetworkopen.2024.31110)
Supplement: Supplement 2. — Data Sharing Statement [file jamanetwopen-e2431110-s002.pdf]

Data Sharing Statement

Markell. Consumer Perceptions of Safety Information in Direct-to-Consumer Print Advertisements for Alzheimer Drugs. *JAMA Netw Open*. Published September 03, 2024. doi:10.1001/jamanetworkopen.2024.31110

Data
Data available: Yes
Data types: Other (please specify)
Additional Information: Data will be available upon request
How to access data: Data will be available upon request - please email jmarkel4@jhu.edu
When available: With publication

Supporting Documents
Document types: None

Additional Information
Who can access the data: Anyone requesting the data
Types of analyses: Any purpose
Mechanisms of data availability: With investigator support/after approval of a proposal
